# Metformin Preserves VE–Cadherin in Choroid Plexus and Attenuates Hydrocephalus via VEGF/VEGFR2/p-Src in an Intraventricular Hemorrhage Rat Model

**DOI:** 10.3390/ijms23158552

**Published:** 2022-08-02

**Authors:** Dan Shen, Xianghua Ye, Jiawen Li, Xiaodi Hao, Luhang Jin, Yujia Jin, Lusha Tong, Feng Gao

**Affiliations:** 1 Department of Neurology, The Second Affiliated Hospital, School of Medicine, Zhejiang University, Hangzhou 310009, China; 21918582@zju.edu.cn (D.S.); xianghua_ye@zju.edu.cn (X.Y.); 12018449@zju.edu.cn (J.L.); 11518198@zju.edu.cn (X.H.); 22018531@zju.edu.cn (L.J.); 22018532@zju.edu.cn (Y.J.); 2 Department of Neurology, Henan Province People’s Hospital, People’s Hospital of Zhengzhou University, Zhengzhou 450000, China

**Keywords:** IVH, hydrocephalus, VE–cadherin, metformin, choroid plexus, VEGF, VEGFR2

## Abstract

Hydrocephalus induced by intraventricular hemorrhage (IVH) is associated with unfavorable prognosis. The increased permeability of choroid plexus and breakdown of the blood–brain barrier (BBB) was reported as a prominent mechanism of IVH-induced hydrocephalus, and vascular endothelial–cadherin (VE–cadherin) was demonstrated to be relevant. Metformin was reported to protect endothelial junction and preserve permeability widely; however, its role in hydrocephalus remains unclear. In this study, the decreased expression of VE–cadherin in the choroid plexus, accompanied with ventricle dilation, was investigated in an IVH rat model induced by intraventricular injection of autologous blood. Metformin treatment ameliorated hydrocephalus and upregulated VE–cadherin expression in choroid plexus meanwhile. We then observed that the internalization of VE–cadherin caused by the activation of vascular endothelial growth factor (VEGF) signaling after IVH was related to the occurrence of hydrocephalus, whereas it can be reversed by metformin treatment. Restraining VEGF signaling by antagonizing VEGFR2 or inhibiting Src phosphorylation increased the expression of VE–cadherin and decreased the severity of hydrocephalus after IVH. Our study demonstrated that the internalization of VE–cadherin via the activation of VEGF signaling may contribute to IVH-induced hydrocephalus, and metformin may be a potential protector via suppressing this pathway.

## 1. Introduction

Intraventricular hemorrhage (IVH) frequently occurs in patients with intracerebral hemorrhage (ICH) and subarachnoid hemorrhage (SAH), and is associated with high morbidity and mortality [1,2,3]. Hydrocephalus, a severe complication of IVH, is recognized as a prominent risk factor of poor prognosis [4,5]. Current research confirms that the obstruction of CSF outflow or reduced reabsorption caused by blood clots blockage, and the increased generation of CSF caused by blood metabolites, are the two main mechanisms of hydrocephalus after IVH [4,6,7,8,9]. However, there are few effective treatments targeting hydrocephalus yet [10], which surges an urgent need to explore other potential mechanisms during the process.

The choroid plexus (ChP) is a highly vascularized tissue that secrets cerebrospinal fluid (CSF) and was suggested as a critical contributor in hydrocephalus after IVH [11]. Together with tight junctions and adherens junctions, ChP constructs the blood–brain barrier (BBB) and prevents paracellular passage [11,12]. Our previous studies discovered that the decrease of vascular endothelial-cadherin (VE–cadherin) in ChP was associated with thrombin-induced hydrocephalus [13]. VE–cadherin, a component of adherens junctions, is essential for vascular integrity and permeability [14,15]. The reduction of VE–cadherin in the central nervous system could lead to brain edema, hydrocephalus, and neuroinflammation for the breakdown of BBB integrity [13,16,17].

It is widely accepted that the breakdown of VE–cadherin in endothelial cells is triggered by vascular endothelial growth factor (VEGF) [14,18]. VEGF, an angiogenic factor regulating angiogenesis and vascular permeability, functions via binding with VEGF receptor 2 (VEGFR2), then results in the VE–cadherin phosphorylation and internalization by a Src-dependent mechanism [14,18,19]. Several studies showed that the expression of VEGF is elevated in neurological disorders [20,21,22,23,24,25]. In the ischemic stroke model, it was found that VEGF destroyed the integrity of the BBB in the acute phase, while playing a reciprocal role as neuroprotection in the recovery phase [21]. Notably, VEGF elevation was reported in patients with hydrocephalus [22,23,24]. Animal models showed that injection of VEGF into the lateral ventricle could cause hydrocephalus in rats, and VEGF inhibitor bevacizumab can restrain the formation of hydrocephalus [25]. However, the effect of VE–cadherin in homologous blood-induced hydrocephalus and whether it is related with the activation of VEGF remains unknown.

Metformin, a most widely prescribed hypoglycemic drug, has multiple effects of anti-inflammation, endothelial protection and, notably, junction protein reservation [26,27,28,29,30]. Zhao et al. discovered that metformin treatment could reverse junction protein reduction in endothelial cell and permeability exacerbation induced by VEGF exposure, which furthers ameliorate tumor-induced edema [31]. Moreover, metformin was reported to attenuate permeability of BBB and thus alleviate brain edema in the ischemia/reperfusion injury and traumatic brain injury of rats [32,33,34]. However, the role of metformin in VE–cadherin expression and then in IVH-induced hydrocephalus remains unknown.

In this study, we aim to explore the role of VE–cadherin internalization mediated by VEGF/VEGFR2/p-Src in IVH-induced hydrocephalus and the mechanisms of metformin’s protection.

## 2. Results

### 2.1. Ventricular Injection of Homologous Blood Caused Hydrocephalus and Downregulation of VE–Cadherin Expression in Choroid Plexus

**Figure 1:**
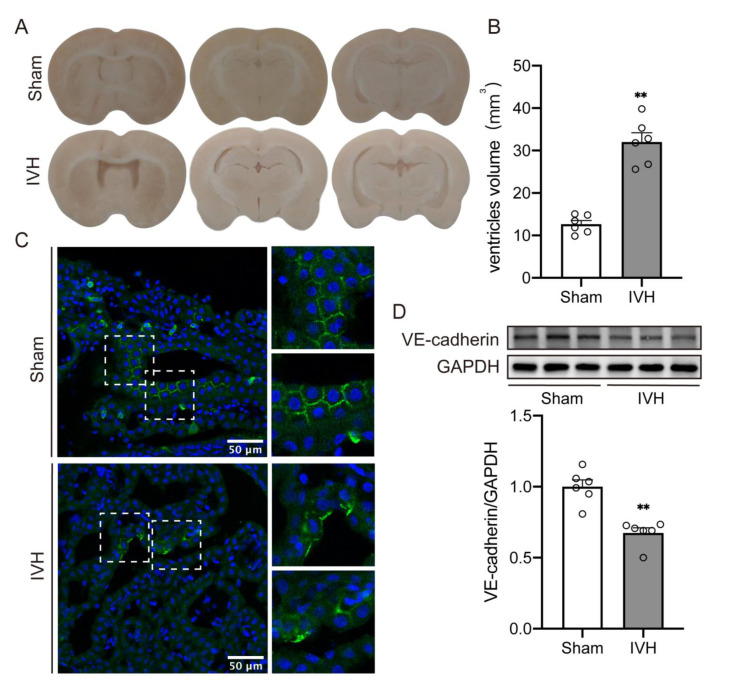
Ventricular injection of homologous blood caused hydrocephalus and downregualtion of choroid plexus VE–cadherin expression (**A**) Frozen coronal brain sections at 7 days after injection of 200 μL of saline or blood into the right lateral ventricle. (**B**) Quantification of lateral ventricle volume from section images, n = 6. (**C**) Immunofluorescence staining of coronal sections showed the expression of VE–cadherin in choroid plexus, n = 4, Scale bar = 50 μm. (**D**) VE–cadherin expression level was assessed by western blot, n = 6. Data were analyzed by *t*-test for comparisons between two groups. Values are means ± SEM, ** *p* < 0.01.

This current study confirms that the injection of homologous blood results in significant ventricular dilation (Figure 1A). Lateral ventricular volumes in IVH rats were significantly larger than in rats receiving the saline injection at 7 days (Figure 1B, 32.02 ± 2.166 vs. 12.65 ± 0.8726 mm^3^ in sham, *p* < 0.01). In order to explore the role of VE–cadherin in IVH-induced hydrocephalus, we investigated the level of VE–cadherin expression in choroid plexus. In the sham group, VE–cadherin was found to be mainly distributed in the borders of choroid plexus endothelial cells, which was significantly declined after IVH (Figure 1C). Western blot equally revealed a reduction of VE–cadherin expression in IVH rats, compared to the sham group (Figure 1D, *p* < 0.01).

### 2.2. Metformin Attenuated the Hydrocepralus and Increased VE–Cadherin Expression in Choroid Plexus after IVH

**Figure 2:**
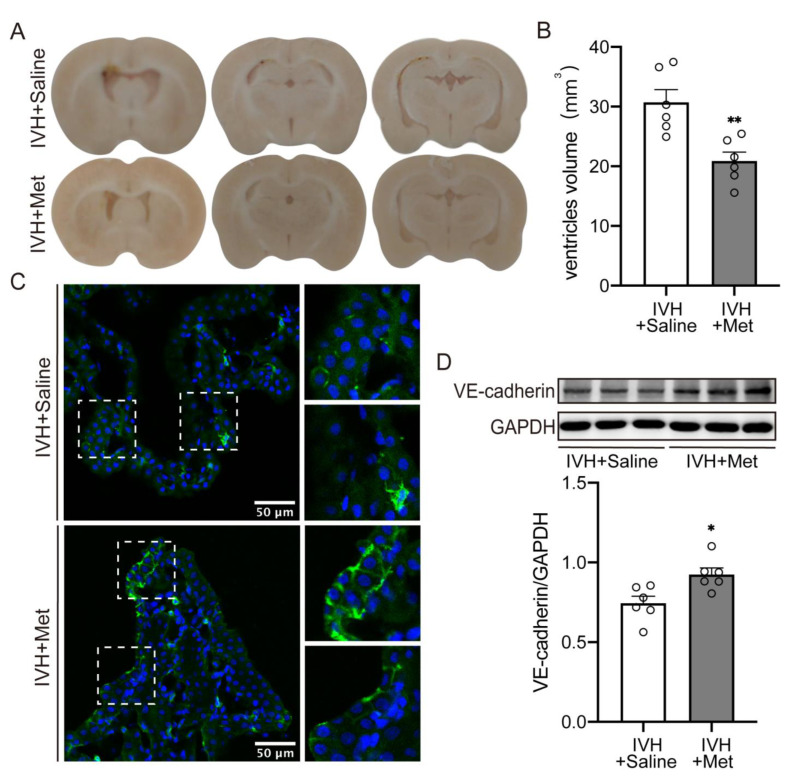
Metformin attenuated the hydrocepralus and increased VE–cadherin expression in choroid plexus after IVH (**A**) Injection of 200 μL of blood into the right lateral ventricle, rats were then treated with saline or metformin for 7 days, examples of frozen coronal brain sections. (**B**) Quantification of lateral ventricle volume from section images, n = 6. (**C**) Immunofluorescence staining of coronal sections showed the expression of VE–cadherin in choroid plexus, n = 4, Scale bar = 50 μm. (**D**) VE–cadherin expression level was assessed by western blot, n = 6. Data were analyzed by *t*-test for comparisons between two groups. Values are means ± SEM, * *p* < 0.05, ** *p* < 0.01.

To examine the mechanisms of metformin’s protection in IVH-induced hydrocephalus, rats were treated with metformin (50 mg/kg, i.p.) or saline continuously for 7 days after IVH. Results showed a significant reduction in hydrocephalus compared to saline injection (Figure 2A,B, 30.72 ± 2.132 vs. 20.88 ± 1.509 mm^3^ in IVH + Saline, *p* < 0.01). In this study, we hypothesized that metformin could attenuate hydrocephalus by enhancing VE–cadherin expression. As immunofluorescence staining and western blot revealed, the increase of VE–cadherin was accompanied with hydrocephalus amelioration after metformin treatment (Figure 2C,D, *p* < 0.05).

### 2.3. Metformin Downregulated the Internalization of VE–Cadherin in Choroid Plexus after IVH

**Figure 3:**
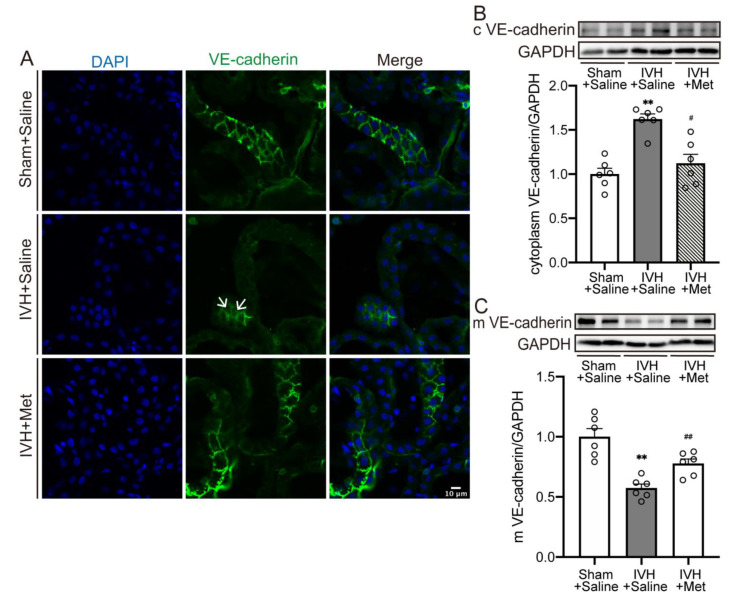
Metformin downregulated the internalization of VE–cadherin in choroid plexus after IVH (**A**) immunofluorescence staining of coronal sections showed the internalization of VE–cadherin, white arrow showed the intracellular fluorescent signal, n = 4, Scale bar = 10 μm. (**B**) Western blot showed the VE–cadherin expression level in cytoplasm, n = 6. (**C**) Western blot showed the VE–cadherin expression level in membrane, n = 6. Data were analyzed by one-way ANOVA with a Tukey’s post hoc test. Values are means ± SEM, ** *p* < 0.01 vs. sham, # *p* < 0.05 vs. IVH + Saline, ## *p* < 0.01 vs. IVH + Saline.

We further evaluated whether the reduction of VE–cadherin was associated with internalization. As immunofluorescence staining showed, scattered fluorescent spots in the cytoplasm were detected at 7 days after IVH, which were diminished by metformin treatment (Figure 3A). Likewise, quantification confirmed a reduction of membrane VE–cadherin in choroid plexus after IVH (Figure 3C, *p* < 0.01), accompanied with an elevation in cytoplasm (Figure 3B, *p* < 0.01), while continuous metformin injection could reverse it (Figure 3B,C, *p* < 0.05). Results indicate the internalization and breakdown of membrane VE–cadherin could be attenuated by metformin.

### 2.4. Metformin Diminished the Upregulation of VEGF Expression after IVH

**Figure 4:**
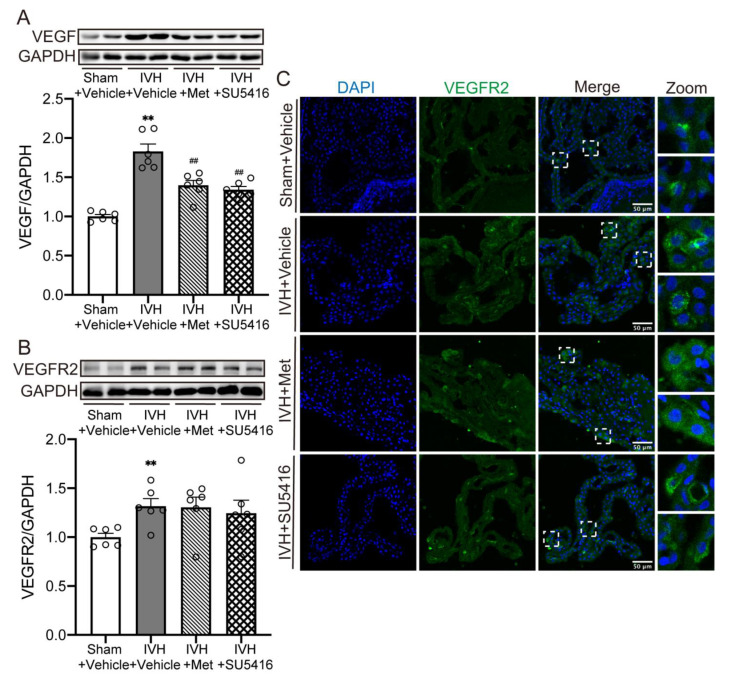
Metformin decreased the upregulated expression of VEGF after IVH (**A**) Western blot showed the VEGF expression level in choroid plexus, n = 6. (**B**) Western blot showed the VEGFR2 expression level in choroid plexus, n = 6. (**C**) immunofluorescence staining of coronal sections showed the expression of VEGFR2 in choroid plexus, n = 4, Scale bar = 50 μm. Data were analyzed by one-way ANOVA with a Tukey’s post hoc test. Values are means ± SEM, ** *p* < 0.01 vs. sham, ## *p* < 0.01 vs. IVH + Vehicle.

Rats were treated with SU5416 (25 mg/kg, Day0 and Day3, i.p.), an inhibitor of VEGFR2, or metformin after IVH. Compared to the sham group, the expression of VEGF (Figure 4A, *p* < 0.01) and VEGFR2 (Figure 4B, *p* < 0.01) significantly increased after IVH. However, inhibitor of VEGFR2 or metformin treatment downregulated the level of VEGF only (Figure 4A, *p* < 0.01), while the impact on VEGFR2 expression was not significant (Figure 4B). This was further investigated using a laser-scanning confocal microscope, indicating an increase in VEGFR2 localization to choroid plexus after IVH (Figure 4C). These results showed that metformin could decline the signal of VEGF with VEGFR2 by decreasing the expression of VEGF after IVH.

### 2.5. Inhibition of VEGFR2/p-Src Attenuated IVH-Induced Hydrocephalus and Escalated the Level of VE–Cadherin

**Figure 5:**
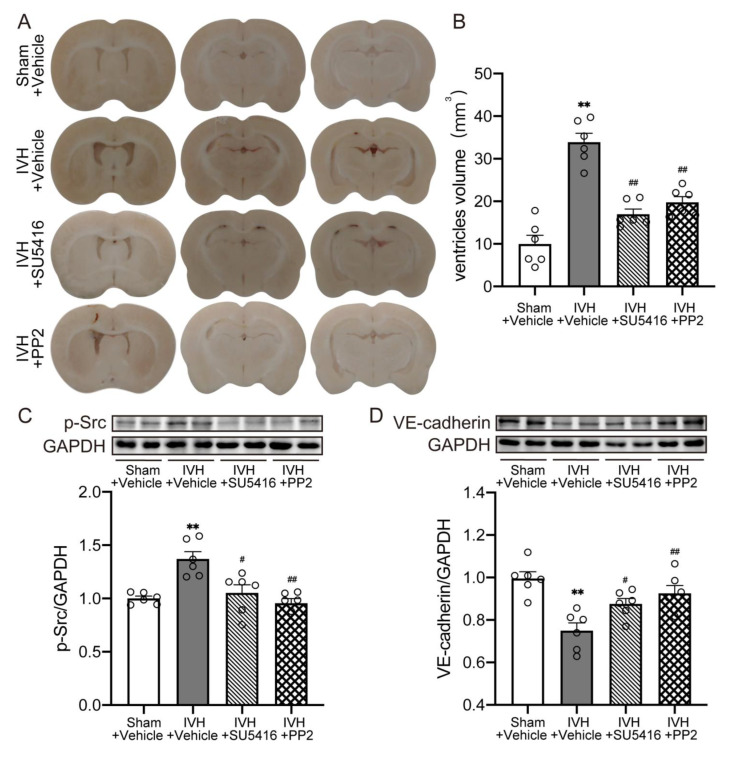
Inhibition of VEGFR2/p-Src attenuated IVH-induced hydrocephalus and increased the level of VE–cadherin (**A**) Frozen coronal brain sections at 7 days after injection of 200 μL of saline or blood into the right lateral ventricle with a treatment of vehicle/SU5416/PP2. (**B**) Quantification of lateral ventricle volume from section images, n = 6. (**C**) p-Src expression level was assessed by western blot, n = 6. (**D**) VE–cadherin expression level was assessed by western blot, n = 6. Data were analyzed by one-way ANOVA with a Tukey’s post hoc test. Values are means ± SEM, ** *p* < 0.01 vs. Sham, # *p* < 0.05 vs. IVH + Vehicle, ## *p* < 0.01 vs. IVH + Vehicle.

We next used the VEGFR2 inhibitor (SU5416, 25 mg/kg, Day0 and Day3, i.p.), Src inhibitor (PP2, 1 mg/kg, Day0 and Day3, i.p.) to explore the downstream mechanism in IVH-induced hydrocephalus. Results revealed that infusing SU5416 or PP2 alleviated ventricular dilation after IVH (Figure 5A,B, *p* < 0.01). Moreover, the increase of p-Src was reversed by inhibiting of VEGFR2 or p-Src (Figure 5C, *p* < 0.05), while the expression of VE–cadherin was upregulated (Figure 5D, *p* < 0.05). This indicated that inhibiting VEGF/VEGFR2/p-Src pathway could increase VE–cadherin expression and then ameliorate IVH-induced hydrocephalus.

Together with previous results, these data demonstrated that metformin may restore the expression of VE–cadherin in choroid plexus after IVH, which is dependent on VEGF signal activation, and thus attenuate hydrocephalus.

## 3. Discussion

This study demonstrates these findings as follows: (1) the internalization and reduction of membrane VE–cadherin in choroid plexus contributed to IVH-induced hydrocephalus; (2) metformin inhibited VEGF/VEGFR2/p-Src pathway activation and reversed the internalization of VE–cadherin, thereby ameliorated IVH-induced hydrocephalus.

Hydrocephalus induced by IVH is associated with unfavorable long-term outcomes [35,36]. The possible mechanisms of hydrocephalus may be attributed to unbalanced CSF-generating and CSF-clearing, in which choroid plexus makes a critical impact [37,38,39,40].The choroid plexus, which is composed of choroid plexus epithelial cells and fenestrated vascular endothelial cells, constructs the BBB and regulates the movement of water and solutes [12,41]. Controlling paracellular and transcellular channels, junction protein can affect the permeability of ChP [42]. Our previous studies discovered the decrease of VE–cadherin in ChP caused BBB destruction and leakage aggravation in thrombin-induced hydrocephalus [13]. Consistent with these findings, we observed intraventricular injection of autologous blood triggered the reduction of VE–cadherin in the choroid plexus, which was responsible for subsequent hydrocephalus.

VE–cadherin is essential for vascular integrity and the restrictive barrier, while the intracellular domain of VE–cadherin can be phosphorylated due to VEGF activation [43,44,45]. The main signal receptor of VEGF, VEGFR2, once be phosphorylated, could regulate the activation of Src kinases, and thus induce VE–cadherin phosphorylation and internalization [18,45,46]. In addition, VEGF signaling reduces the linkage of VE–cadherin and associated proteins, such as p120-catenin and β-catenin, aggravates the endocytosis of VE–cadherin [47,48]. Besides being partially degraded, the intracellular VE–cadherin can relocate to the cell membrane after removal of VEGF stimulation [14,18].

Some studies revealed that VEGF was increased after stroke or hydrocephalus. VEGF is secreted by neurons, pericytes, astrocytes, microglia, macrophages and abundantly by choroid plexus in nervous system, while VEGFR2 is significantly expressed on ependymal cells, vascular endothelium, and ChP in the ventricle [19,49,50,51]. Upon the activation of VEGF, the choroid plexus vascular endothelial fenestration is induced to promote the water and solutes transport from blood to ventricle [50,52]. Our results show that the expression of VEGF and VEGFR2 in ChP increased after IVH. Besides, further investigation revealed that the expression of VE–cadherin decreased in the cell membrane, while increased in the cytoplasm, indicating the increased internalization after IVH. The maldistribution of VE–cadherin in ChP resulted in the destruction of intercellular junction, the aggravation of fenestration and the breakdown of BBB, which contributed to hydrocephalus. Moreover, barrier disruption caused astrocytes and microglia/macrophages activation in periventricular area, upregulated VEGF expression, and then aggravated barrier breakdown [51]. Obstruction caused by hematoma compounds may also function in VEGF accumulation and subsequent VE–cadherin internalization.

Furthermore, specific antagonism of VEGFR2 after IVH reduced the phosphorylation of downstream Src in the choroid plexus and upregulated the expression of VE–cadherin. In accordance with this finding, Src kinase inhibitor also escalated the VE–cadherin expression and thus alleviated the occurrence of hydrocephalus. Our results corroborate that the activation of VEGF/VEGFR2 signaling in the choroid plexus after IVH results in increased VE–cadherin internalization, leading to the occurrence of hydrocephalus.

Metformin is a most widely prescribed hypoglycemic drug. However, the glycemic value of rats receiving the metformin treatment was not staticticaly different from saline treatment in our experiment. Metformin also was known to protect endothelial barrier and decrease permeability via junction reinforcement, anti-inflammation, anti-autophagy, and anti-oxidant [53,54,55,56]. Moreover, some studies revealed that metformin treatment could protect BBB [57]. In a rat model of middle cerebral artery occlusion, metformin was found to decrease BBB permeability via downregulating intercellular adhesion molecule-1 (ICAM-1) and inflammation cytokines [58]. Furthermore, metformin was reported to prevent endothelial permeability induced by VEGF or hypoxia treatment, and attenuated gliama-induced brain edema [31]. Notably, some studies uncovered that metformin could increase endothelial junction and downregulate the expression of VEGF [29,59,60]. Consistent with these findings, we found that metformin could attenuate IVH-induced hydrocephalus.

In our study, we observed that metformin upregulated VE–cadherin expression in choroid plexus, and then ameliorated ventricle dilation. Moreover, the internalization was reversed after metformin continuous treatment, accompanied by VEGF reduction. In contrast, a poor impact was found on VEGFR2 expression. Restraining VEGF signaling by antagonizing VEGFR2 or inhibiting Src phosphorylation increased the expression of VE–cadherin and the severity of hydrocephalus after IVH. Together with these findings, metformin could reduce the expression of VEGF, thus diminishing the activation of VEGF/VEGFR2/p-Src pathway, restored the membrane VE–cadherin expression in choroid plexus, and then, in this way, attenuated hydrocephalus.

However, there is also considerable evidence on other mechanisms of metformin in hydrocephalus protection. For example, tight junction proteins, such as ZO-1 and claudin-5 of ChP, may also contribute to BBB permeability. Metformin was reported to decrease BBB permeability via increasing claudin-5 expression and restoring ZO-1 distribution [61]. Besides, activated by NF-κB dependent inflammatory signal, the potassium cotransporter in ChP, NKCC1, was reported to be upregulated after IVH, accompanied with the CSF hypersecretion, which may also make sense in metformin’s protection in IVH-induced hydrocephalus [62]. Additionally, the brain parenchymal system controlling glymphatic CSF-ISF exchange was reported as an another source of CSF production [63]. Without fenestrations, the astrocyte aquaporin 4 (AQP4) system promotes water influx into the peri-capillary Virchow-Robin space (VRS) and regulates the CSF circulation together with ChP [64]. Zhao et al. found that metformin treatment could reduce the expression of AQP4 on astrocytes and ameliorate tumor-induced edema [31]. Whether the glymphatic system was associated with the function of metformin in IVH remains unknown. These potential mechanisms need to be further evaluated in our future research.

## 4. Materials and Methods

### 4.1. Animals Model

Animal protocols were approved by the Zhejiang University Animal Experimentation Committee on the Use and Care of Animals. A total of 140 male Sprague–Dawley rats (3-month old, Zhejiang University Laboratories, Zhejiang, China) were used in this study, at the weight of 280–320 g. Animals were anesthetized with pentobarbital (50 mg/kg intraperitoneally (i.p.)) and were positioned in a stereotaxic frame (MICRO2T, World Precision Instruments, Sarasota, Florida, USA). A cranial burr hole (1 mm) was drilled 0.6 mm posterior, 1.6 mm lateral to the bregma, and a 26-gauge needle was inserted 4.5 mm ventral into the right lateral ventricle through the hole. A total of 200 µL homologous blood or saline was injected over 15 min using a microinfusion pump (MICRO2T, World Precision Instruments, Sarasota, Florida, USA). The needle remained for 10 min, and was then gently removed. The burr hole was filled with bone wax, and the skin incision was sutured.

### 4.2. Experimental Groups

**Figure 6:**
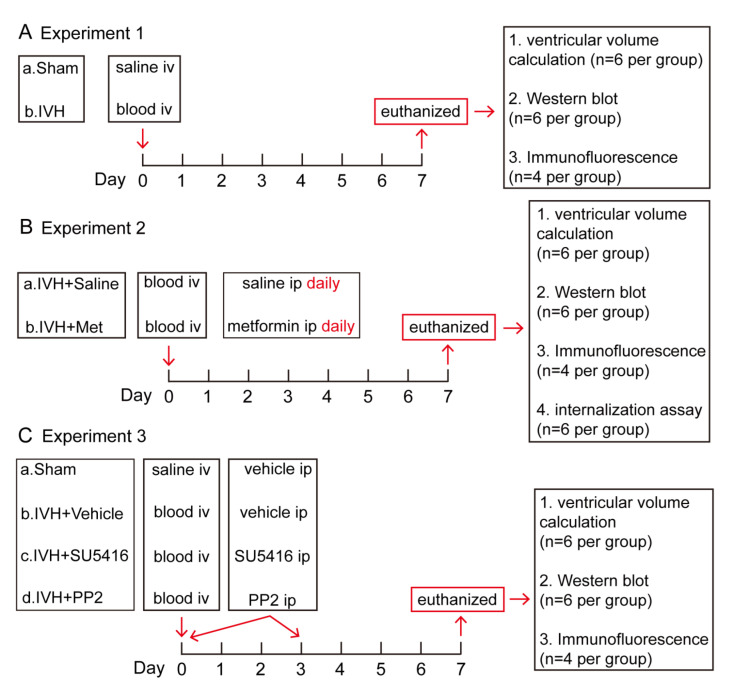
Experimental designs. (**A**) Experiment 1 was designed to investigate ventricular dilation and the VE–cadherin expression after IVH. (**B**) Experiment 2 was designed to explore the mechanism of metformin’s protection in IVH-induced hydrocephalus. (**C**) Experiment 3 was designed to explore the mechanism of VEGF/VEGFR2/p-Src pathway in hydrocephalus.

Briefly, this study has three parts. First, rats were randomly divided into two groups (sham, IVH). The IVH group received an injection of 200 μL homologous blood while the sham group received the same volume of saline into the right lateral ventricle. Rats were euthanized at day 7, the brains were used for ventricular volume calculation (n = 6 per group), western blot (n = 6 per group) and brain histology (n = 4 per group). Second, rats were treated with metformin [65,66] (Abcam, 50 mg/kg, i.p.) or saline (equal volume, i.p.) daily for the following 7 days after IVH, then were euthanized at Day 7. The brains were used for ventricular volume calculation (n = 6 per group), western blot (n = 6 per group), internalization assay (n = 6 per group) and brain histology (n = 4 per group). Third, rats were divided into sham, IVH + Vehicle (2% dimethyl sulfoxide in saline), IVH + VEGFR2 inhibitor (SU5416, MCE, 25 mg/kg, i.p.) and IVH + p-Src inhibitor (PP2, Abcam, 1 mg/kg, i.p.). SU5416 or PP2 were injected at day 0 and day 3 [13,67,68]. At day 7, rats were euthanized and the brains were used for ventricular volume calculation (n = 6 per groups), western blot (n = 6 per groups) and brain histology (n = 4 per groups) (Figure 6).

### 4.3. Ventricular Volume Analysis

Rats were perfused with 4% paraformaldehyde in 0.1 mol/L phosphate-buffered saline (pH 7.4). The brains were removed and kept in 4% paraformaldehyde for 24 h and then protected in 30% sucrose. Brains were embedded in optimal cutting temperature compound and 200 μm thick slices cut using a cryostat (LEICA, CM3050S). In the meantime, we took high-resolution pictures of serial coronal sections using same camera (200 μm apart, Logitech) positioning and external lighting [62]. Bilateral ventricles from frontal horns to the fourth ventricle were outlined and measured using Image J software [13]. Pixels were converted to area, and ventricular volume (mm^3^) was calculated by summing the ventricle areas over all sections and multiplying by the distance (200 μm apart). All image analysis was performed by a blinded investigator. All statistical analysis were completed in GraphPad Prism 8 (GraphPad software).

### 4.4. Immunofluorescence Staining

Rats were perfused with 4% paraformaldehyde in 0.1 mol/L phosphate-buffered saline (pH 7.4). The brains were removed and kept in 4% paraformaldehyde for 24 h and then dehydrated in 30% sucrose for 2 to 3 days at 4 °C. Brains were embedded in optimal cutting temperature compound and 18-μm thick slices cut (coronal) using a cryostat (CM3050S, LEICA, Wetzlar, Germany). Sections were blocked 5% donkey serum albumin for 2 h at room temperature and then incubated with mouse anti-VE–cadherin (1:50 dilution, Santa Cruz Biotechnology, Dallas, TX, USA) or rabbit anti-VEGFR2 (1:800 dilution, Cell Signaling Technology, Danvers, MA, USA) at 4 °C overnight. After washed three times with PBS, sections were incubated with appropriate secondary antibodies (1:400 dilution, donkey anti-mouse Alexa Fluor 488, donkey anti-rabbit Alexa Fluor 488, Abcam, Cambridge, UK) for 1 h at room temperature. Then, using a fluoroshield mounting medium with DAPI (Abcam, Cambridge, UK), evaluation was performed under a confocal laser scanning microscope (FV3000, Olympus, Tokyo, Japan). Images were collected randomly from five fields and experiments were repeated in at least three times.

### 4.5. Western Blot

Brain tissue was homogenized (Diax 900, Heidolph, Schwabach, Germany) in a western sample buffer. The lysate was centrifuged at 13,600× *g* for 30 min at 4 °C, and then the supernatant was collected. Proteins concentration was determined by the BCA protein assay kit (Pierce, Thermo Fisher Scientific, Waltham, MA, USA). Proteins were separated using SDS-polyacrylamide gel electrophoresis (BIO-RAD, Hercules, CA, USA) and transferred to the nitrocellulose membrane (Merck Millipore, Burlington, MA, USA). After blocking with 5% nonfat milk for 1 h, the membranes were incubated with primary antibodies anti-VE–cadherin (1:200 dilution, Santa Cruz), anti-p-Src (1:1000 dilution, Cell Signaling Technology, Danvers, MA, USA), anti-VEGFA (1:1000 dilution, Abcam, Cambridge, UK), anti-VEGFR2 (1:1000 dilution, Cell Signaling Technology, Danvers, MA, USA) at 4 °C overnight. Then, the membranes were reacted with antibodies against rabbit IgG (1:3000, Affinity Biosciences, OH, USA) or mouse IgG (1:3000, Affinity Biosciences, OH, USA) for 2 h at room temperature. Protein signals were visualized using a chemiluminescence detection system (Tanon, Shanghai, China), and bands were analyzed with Image J software.

For VE–cadherin internalization, the proteins in cell membrane and cytoplasm were isolated by the membrane protein extraction kit (P0033, Beyotime, Jiangsu, China). Briefly, brain tissue was homogenized in membrane protein extraction reagent A, and incubated for 10–15 min. The lysate was centrifuged at 3000× *g* for 10 min at 4 °C, and then the supernatant was collected. After that, the obtained supernatant was centrifuged at 14,000× *g* for 30 min, and the supernatant was extracted as the cytoplasmic protein solution. A membrane protein extraction reagent B was added to the sediment and incubated for 5–10 min. After centrifugation at 14,000× *g* for 10 min at 4 °C, the supernatant was collected as the cell membrane protein solution. Then, proteins were studied with western blot as described above.

### 4.6. Statistical Analysis

Values was given in means ± SD. The data was analyzed with *t*-test and one-way ANOVA with a Tukey’s post hoc test. All statistics were completed in GraphPad Prism 8 (GraphPad software). Differences were considered significant at *p* < 0.05.

## Data Availability

The data used and/or analyzed during the current study are available from the corresponding author on reasonable request.
